# A Case of Encysted Tumor Extirpated

**Published:** 1852-11

**Authors:** M. J. Bray

**Affiliations:** Evansville, Indiana


ART. III.—*A Case of Encysted Tumor Extirpated.* By. M. J, BRAY, of Evansville, Ia.

Mr. James Smith, residing in the country, came to Evansville to have a large tumor removed from the neck. He had, naturally, a good constitution, but at this time, and for two or three months previously, he had suffered from the irritable effects of the tumor. He was somewhat emaciated and reduced in strength, and had a slight fever every night, which disturbed his rest.

He was about forty years of age. The tumor made its appearance sixteen years ago, and has since gradually increased in size. Its base was composed of conglomerations of adipose tissue, but the middle and apex appeared to be encysted.

It had become complicated in its organization in consequence of repeated attacks of inflammation. It was situated on the head and neck, occupying the whole of one side, extending from the spinous processes of the cervical vertebra to the median line of the neck in front,

It measured twenty-six inches in circumference, and thirty-one inches from its base on one side, over its apex, to the other, and weighed, after it was removed, twelve pounds.

The operation lasted three quarters of an hour. The arteries were ligated as soon as they were divided.—Seven or eight required the ligature, and the strength of the patient was supported by the liberal administration of brandy, given in small doses.

After the tumor was removed, the flaps of the wound were placed in coaptation, and secured in the usual way. A large proportion of it united by the first intention, and in ten days from the time of the operation the patient was able to return home.

I think this tumor is as large as any, if not the largest, which has been successfully extirpated in the United States.

EVANSVILLE, IA., October, 1852.

